# Extra-axial isolated cerebral varix misdiagnosed as convexity meningioma: A case report and review of literatures

**DOI:** 10.1097/MD.0000000000004047

**Published:** 2016-07-01

**Authors:** Zhi-Gang Tan, Qian Zhou, Yan Cui, Lei Yi, Yian Ouyang, Yugang Jiang

**Affiliations:** Department of Neurosurgery, The Second Xiangya Hospital of Central South University (CSU), Changsha, Hunan, China.

**Keywords:** convexity meningioma, differential diagnosis, isolated cerebral varix, treatment

## Abstract

Isolated cerebral varix is a rare cerebrovascular anomaly, which is easily misdiagnosed as other brain tumors.

A 59-year-old female patient with noncontributory medical history presented with headache and insomnia for the last 2 months. Upon admission, her neurological examination was unremarkable. Magnetic resonance imaging revealed a well-demarcated extra medullary mass, 11 × 11 mm in size, within the subdural space at the right frontal lobe. The lesion was initially interpreted as a convexity meningioma. After conducting a craniotomy on the patient, an extra-axial varix was exposed and resected subsequently. The patient's headache was resolved soon after surgery and charged without neurologic sequelae.

Extra-axial isolated cerebral varix is mimicking convexity meningioma on MR images and should be considered as a differential diagnosis. The focal erosion in the inner table of the skull could be an important character of extra-axial isolated cerebral varix. An extremely round shape and smooth contour of the lesion was another important character. Isolated cerebral varix is rare vascular lesion that is treated surgically in the case of rupture or compression of adjacent structures. The information obtained with noninvasive imaging techniques should include CTA to make a clinical decision.

## 1 Introduction

Cerebral varices are mostly associated with Galen aneurysms. In other cases, they are observed in arteriovenous malformation (AVM ,)^[1,2]^ arteriovenous fistula (AVF),^[3,4]^ and developmental venous anomalies (DVA).^[5–7]^ However, an isolated cerebral varix is rarely documented phenomenon, which may present as mass lesion on computed tomography (CT) or magnetic resonance imaging (MRI). Some cases, the isolated cerebral varix, could be described as incidental finding without any neurological symptoms. Due to its minor clinical manifestations, it is easily misinterpreted as cystic or tumoral lesion, which makes its diagnosis challenging.

## 2 Ethical approval and consent

Ethical approval was given by the medical ethics committee of The Second Xiangya Hospital of Central South University. The patient signed the necessary documents to consent to the use of his data for teaching and publication.

## 3 Case report

**Figure 1:**
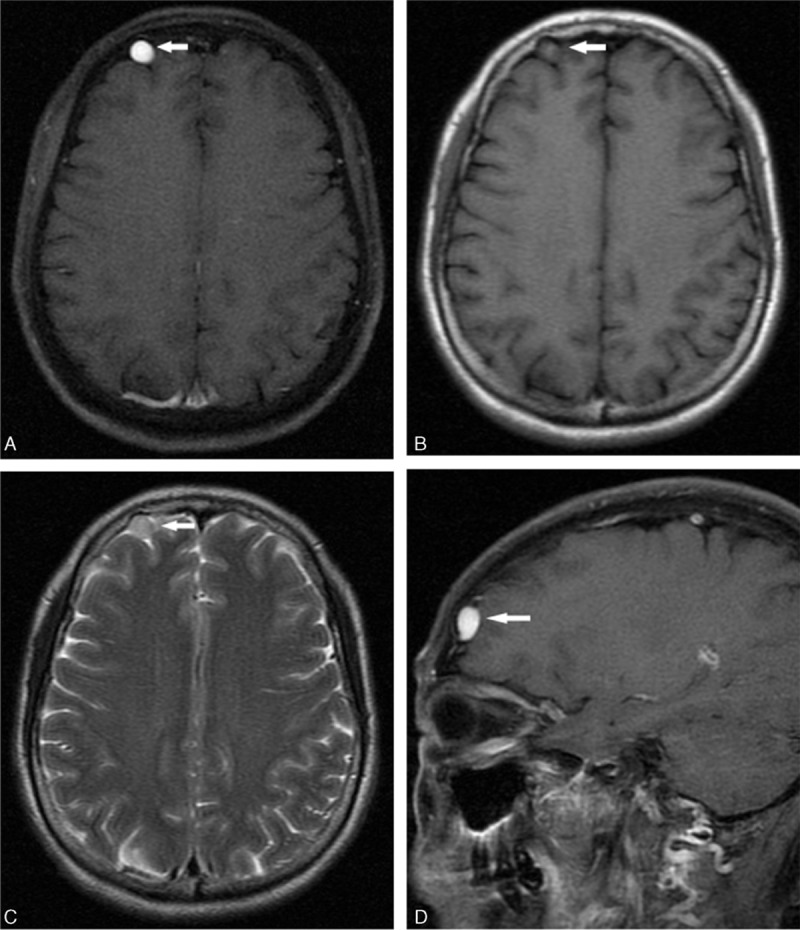
Preoperative magnetic resonance images postcontrast images revealed a rounded lesion on the axial T1-weighted image (arrow, A) and the spindle-shaped on sagittal image (arrow, D), with homogeneous-marked enhancement in the right frontal convexity. It is isointense with gray matter on the T1-wighted image (arrow, B) and had slightly hyperintensity on the T2-wighted image (arrow, C).

A 59-year-old female without relevant medical history complained of 2 months of right, unilateral, intermittent headache, associated with insomnia that improved with nothing. A detailed neurological examination was performed and revealed no deficit. A plain and enhanced MR scan was performed and revealed a well-defined extra-axial round lesion with clear border located adjacent to the right frontal convexity (Fig. 1A). The abnormality was showing isointense with gray matter on T1-WI images (Fig. 1B) and slightly hyperintensity on T2-WI images (Fig. 1C). Intense homogeneous enhancement after intravenous contrast administration was observed (Fig. 1D). Flow voids were absent. Other abnormal signals in the cerebral parenchyma were not demonstrated on MRI. Due to the unspecific clinical manifestations, convexity location, MR signal intensity, and enhancement characteristics, the diagnosis of an extra-axial convexity meningioma was made.

**Figure 2:**
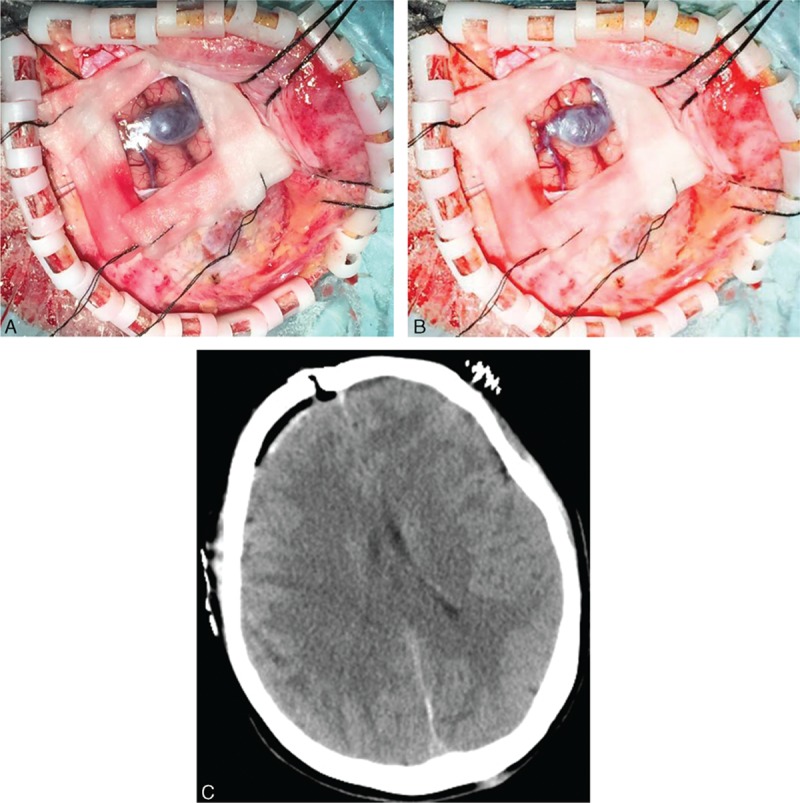
Intraoperative exposure of the isolate cerebral varix and postoperative CT image. A focal dilatation of the convexity vein was found. It has the filling stage (A) and the loose stage (B), which prompts the press on the surrounding cortex, dura and the inner table of skull. No signs of venous congestion or brain tissue edema were observed in the CT image 24 h after the surgery (C). CT, computed tomography.

A craniotomy was operated on the patient. At surgery, a 11-mm spindle-shaped, pliable structure was found. The structure, which pressed on the surrounding cortex, dura and the inner table of skull, was observed with filling (Fig. 2A) and loose stage (Fig. 2B). There was no attachment found between the focal dilation and adjacent dura mater. Both sides of the dilation were normal veins. No arterial feeders were found, and no neural tissues were involved. Conclusion was made as an isolated cerebral varix instead of a convexity meningioma. The isolated cerebral varix was resected entirely. Computed tomography 1 day after the surgery was performed, and no signs of venous congestion or brain tissue edema were showed (Fig. 2C). The patient's headache was resolved soon after surgery and charged without neurologic sequelae. As a result, surgical treatment achieved complete resolution of the headache and insomnia in 1-year follow-up.

## 4 Discussion

### 4.1 Clinical manifestations

Isolated cerebral varices are described as a focal dilatation of a single vein, involving no neural tissue and other vessels anomaly.^[8]^ Most of them are clinic silent and commonly found incidentally. Among the reported symptomatic cases, hemorrhage, thrombosis, seizure, and mass effect are the most common manifestations.^[9]^ Histological investigation showed that no signs of thickened or fibrotic intima were observed in the varix.^[10]^ Roda et al reported a case of a cerebrovascular varix located in the right lateral ventricle. The patient suffered intraventricular and subarachnoid hemorrhages caused by the isolated cerebral varix.^[11]^

Blood in isolate cerebral varices are prone to form turbulence and thrombus. Kondo et al introduced an epileptic patient caused by thrombosis of varix. The patient was well diagnosed as isolated cerebral varix and recommended regular follow-up. By 2 months later, she developed seizure of her left arm. The following CT scan and angiography demonstrated an acute thrombosis of the varix.^[8]^ Furthermore, as for other brain tumors, a large varix may produce symptoms from compression of adjacent structures. Inoue et al first reported a case of trigeminal neuralgia caused by compression from an isolated varix.^[12]^ Patient's plainness was removed immediately after the surgeon transposed the varix with its parent vein from the nerve. Isolated varix in brain lack clinical symptomatic specificities. Physicians need supplementary examinations to provide diagnosis clues and make the clinic decisions.

### 4.2 Radiological characteristic

Symptomatic patients may first receive a CT scan. CT images reveal isolated cerebral varix as isodense lesion precontrast and homogeneous-marked enhancement lesion postcontrast.^[13]^ Shibata et al reported a varix with a thin circular enhancement postcontrast in CT images.^[9]^ Tanohata et al,^[13]^ Dietrich and Forsting ,^[14]^ and Hoell et al^[10]^ reported skull erosion adjacent to varices in their cases. In our case, we also find compression erosion in the inner table of skull. MR scan described isolated cerebral varices as extra-axial, cystic, and well-circumscribed lesions that are isointense with gray matter on noncontrast MR images and exhibit homogeneous-marked enhancement on contrast MR images. Sometimes 2 ends of isolate cerebral varix shows significant enhancement on contrast MR images, mimicking the “dura tail sign”. The lesion in CT or MR images is always well demarcated and spindle-shaped with clear border and smooth contours. A flow void phenomenon in MRI is expected, but not necessary. MR venography (MRV) and digital subtraction angiography (DSA) are recommended for they can reveal detailed venous anatomy of the varix, which are helpful to make conclusive diagnosis. However, it may be flow void phenomenon and angiographically negative for thrombosed varices.^[8,15]^ At this time, the hyperintensity signal on both T1-WI and T2-WI images would be observed.

### 4.3 Differential diagnosis

Isolated cerebral varices can be classified into 2 categories according to their locations: extra-axial and intra-axial. Isolated cerebral varix is easily misdiagnosed as other brain tumors, especially for patients without the presence of a subarachnoid hemorrhage or intracerebral hematoma. Extra-axial isolated cerebral varix mimics convexity meningioma on MR images. They are similar in several aspects, including the MR signal intensity, enhancement characteristics, and even the “dura tail sign,” which easily lead to imaging misdiagnosis.^[16]^ Two features may be helpful in differential diagnosis. One of the features is the detection of focal erosion of the skull on CT images, which seems to be a helpful pattern to distinguish the varix from a meningioma that usually goes with skull hypertrophy.^[10]^ On the other hand, when lesion with extremely round shape and smooth contour was presented on CT or MR images, a vascular origin lesion should be considered. Patients may be free from unnecessary craniotomy for correct diagnosis. For patients whose preliminary diagnosis is convexity meningioma, if their CT/MRI images possessed either feature above, brain vessel MRV was recommended to exclude isolated cerebral varix.

Intra-axial isolated cerebral varices are completely surrounded by parenchyma. The CT and MR appearance may stimulate other vascular anomalies. Kelly et al^[15]^ described a cystic mass within the deep left temporal lobe. Hyperintense was showed on both T1-WI and T2-WI images companied with hypointense on hemosiderin rim. This observation was initially suspected to be a neoplasm, such as cystic astrocytoma, abscess, or thrombosed aneurysm and was finally confirmed to be an intra-axial isolated cerebral varix by operation and pathology. Mouthuy et al provided a case of an isolated cerebral varix stimulating a cavernous angioma.^[17]^ In their case, the contrast CT scan revealed a lesion located in the left occipital lobe with circular enhancement. The MR appearance was extremely similar to Kelly's case. DSA is recommended to distinguish isolated cerebral varix from other common vascular anomalies.

### 4.4 Treatment strategies

For symptomatic patients whose complaints are associated with isolated cerebral varices, resection is recommended. To evaluate the feasibility of the resection, a DSA evaluation of the draining area of the feeding vein preoperative and a temporary blocking of the feeding vessel for 15 minutes intraoperative should be done before excision.^[10]^

However, in most cases, the cerebral varices remain clinically silent and commonly found incidentally. For these varices, medical approaches are quite conflicting. Some clinicians recommended close outpatient observation and regular follow-up.^[9,16]^ Reasons could be the balance between slight clinical manifestation and the high risk of craniotomy under general anesthesia. However, during outpatient observation period, hemorrhage, thrombosis, and seizures may develop, which may be potentially life threatening. In Kondo et al's case, the patient developed acute thrombosis of the varix during outpatient observation.^[8]^ In our opinion, neurosurgeons should be more aggressive about these varices. Different strategies between the nonsymptomatic extra-axial and intra-axial isolated cerebral varices should be taken. For nonsymptomatic extra-axial isolated cerebral varices, their feeding veins are superficial and the surgery is relative simple. Considered potentially life-threatening complications, we suggest early surgical interventions. For intra-axial isolated cerebral varices, the location and size of the lesion, patient's age, and other factors should be taken into account to decide whether surgical intervention or not.

## 5 Conclusion

Isolated cerebral varix is extremely rare and can be classified into extra-axial isolated cerebral varix and intra-axial isolated cerebral varix. Extra-axial isolated cerebral varix has similar location and MR appearance with convexity meningioma, which is easily misdiagnosed. MR angiography and DSA are helpful in differential diagnosis. There is no unified view on treatment of these venous anomalies. Hemorrhage, thrombosis, and seizures may develop during outpatient observation period, which may be potentially life threatening. Surgical approaches are feasible after preoperative and intraoperative evaluation. Early resection of symptomatic extra-axial isolated cerebral varix is beneficial.
